# Costs of current and improved meningitis disease surveillance in Chad

**DOI:** 10.1186/1472-6963-14-S2-P51

**Published:** 2014-07-07

**Authors:** Ulla Griffiths, Ngozi Erondu

**Affiliations:** 1 Department of Global Health and Development, London School of Hygiene and Tropical Medicine, London, UK

## Background

Meningococcal meningitis has been a terrifying public health threat in countries of the Sahel and sub-Sahel over the past 100 years. This geographical area has been named the “African meningitis belt”. Older children and young adults are particularly at risk during epidemics. The mortality rate is usually 5% - 10%. A serogroup A meningococcal conjugate vaccine was licensed in 2009. Mass campaigns with MenAfriVac^®^ covering 1-29 years old have been conducted in several countries with assistance from the GAVI Alliance. Long term health impacts of MenAfriVac^®^ can only be determined if strong disease surveillance is in place. The study objective was to estimate the costs of meningitis surveillance in Chad and determine resources needed for implementing district case based surveillance.

## Materials and methods

Data were collected for 2012. The ingredient approach to costing was used. Data were collected from the national laboratory and seven districts. In each district, three primary care facilities and one district laboratory was included. Resource utilization data were collected from interviews. Resources were categorized according to core surveillance functions (detect, report, analysis, feedback, investigation, and response), and support functions (training, supervision, communication, and co-ordination). Unit costs were collected from financial records of Ministry of Health and international partners. An operational standard was developed for modeling the incremental costs of upgrading the system.

## Results

Optimal surveillance was severely hampered by limited resources. One district laboratory had not been able to conduct any analysis of cerebrospinal fluid (CSF) due to lack of supplies. In 14 of the facilities staff was qualified to perform lumbar puncture on patients with suspected meningitis; patients were referred to district hospitals in the remaining seven facilities. In three of the districts, no meningitis cases were reported during 2012. In the other four, reported cases varied between 43 and 232, equivalent to between 11 and 89 per 100,000 populations. 11% of CSF was sent to the national laboratory for confirmation.

Costs per detected case amounted to US$ 49, with costs of lumbar puncture comprising 43% and laboratory analysis 41% of total costs. In facilities with no detected cases, recourses spent on surveillance were marginal.

## Conclusions

Standard procedures for meningitis surveillance are missing due to lack of supplies at health facilities and laboratories and due to inadequate training of staff. While investments are needed across the system, these are only likely to be sustainable if activities are part of integrated disease surveillance.

